# Prevalent Human Gut Bacteria Hydrolyse and Metabolise Important Food-Derived Mycotoxins and Masked Mycotoxins

**DOI:** 10.3390/toxins12100654

**Published:** 2020-10-13

**Authors:** Noshin Daud, Valerie Currie, Gary Duncan, Freda Farquharson, Tomoya Yoshinari, Petra Louis, Silvia W. Gratz

**Affiliations:** 1 Rowett Institute, University of Aberdeen, Foresterhill, Aberdeen AB25 2ZD, UK; r01nd17@abdn.ac.uk (N.D.); v.currie@abdn.ac.uk (V.C.); gary.duncan@abdn.ac.uk (G.D.); f.farquharson@abdn.ac.uk (F.F.); p.louis@abdn.ac.uk (P.L.); 2 Division of Microbiology, National Institute of Health Sciences, 3-25-26 Tonomachi, Kawasaki-ku, Kawasaki-shi, Kanagawa 210-9501, Japan; t-yoshinari@nihs.go.jp

**Keywords:** mycotoxin-glucosides, trichothecenes, gut microbiota, microbiome, release, de-acetylation

## Abstract

Mycotoxins are important food contaminants that commonly co-occur with modified mycotoxins such as mycotoxin-glucosides in contaminated cereal grains. These masked mycotoxins are less toxic, but their breakdown and release of unconjugated mycotoxins has been shown by mixed gut microbiota of humans and animals. The role of different bacteria in hydrolysing mycotoxin-glucosides is unknown, and this study therefore investigated fourteen strains of human gut bacteria for their ability to break down masked mycotoxins. Individual bacterial strains were incubated anaerobically with masked mycotoxins (deoxynivalenol-3-β-glucoside, DON-Glc; nivalenol-3-β-glucoside, NIV-Glc; HT-2-β-glucoside, HT-2-Glc; diacetoxyscirpenol-α-glucoside, DAS-Glc), or unconjugated mycotoxins (DON, NIV, HT-2, T-2, and DAS) for up to 48 h. Bacterial growth, hydrolysis of mycotoxin-glucosides and further metabolism of mycotoxins were assessed. We found no impact of any mycotoxin on bacterial growth. We have demonstrated that *Butyrivibrio fibrisolvens*, *Roseburia intestinalis* and *Eubacterium rectale* hydrolyse DON-Glc, HT-2 Glc, and NIV-Glc efficiently and have confirmed this activity in *Bifidobacterium adolescentis* and *Lactiplantibacillus plantarum* (DON-Glc only). *Prevotella copri* and *B. fibrisolvens* efficiently de-acetylated T-2 and DAS, but none of the bacteria were capable of de-epoxydation or hydrolysis of α-glucosides. In summary we have identified key bacteria involved in hydrolysing mycotoxin-glucosides and de-acetylating type A trichothecenes in the human gut.

## 1. Introduction

Mycotoxins are toxic secondary metabolites of fungi and are frequently found in a variety of agricultural crops such as cereals, fruits, and nuts. Mycotoxin contamination of crops and their carry over into the human food chain are of great concern as they are potent toxins and their toxicities may contribute to adverse health effects in humans [1]. In response to fungal infection and mycotoxin production, several cereal plants have been found to transform mycotoxins into mycotoxin metabolites. Amongst those metabolites, sugar conjugates or masked mycotoxins have been identified and their co-occurrence with parent mycotoxins in cereals has been confirmed [2,3,4]. Deoxynivalenol-3-β-glucoside (DON-Glc) is the most studied masked mycotoxins, but masked forms of other trichothecenes, zearalenone, and fumonisin have also been reported. Resulting from the prevalence of these masked mycotoxins in foods it is paramount to understand their fate in the human gut and their potential contribution to toxicity. Over the last decade several studies have assessed the fate of DON-Glc and other masked mycotoxins under gastrointestinal conditions in vitro and in vivo, and most studies have found masked mycotoxins to be stable towards small intestinal digestion and not to be absorbed intact [5]. However, microbial hydrolysis of masked mycotoxins by human gut microbiota is well described in vitro [6,7,8,9,10] and further confirmed in pig microbiota [11,12]. Furthermore, the release of DON from a dose of DON-Glc and subsequent absorption and urinary excretion have recently been confirmed to occur in vivo in humans [13]. Individual differences between stool samples from different donors have been reported in the kinetics of mycotoxin release from masked forms and different efficiencies for various types of masked mycotoxins are also evident [7]. Hence, the role of the complex microbiome in the hydrolysis and further metabolism of different masked mycotoxins is poorly understood.

The human colon is colonized by a diverse bacterial population comprising the three most abundant phyla Firmicutes, Bacteroidetes, and Actinobacteria followed by Verrucomicrobia, Proteobacteria, Fusobacteria, and Cyanobacteria [14]. This diverse population of the human colon microbiota plays an important role in host health [15] and its contribution to nutrition and metabolism is well documented [16]. The human colon microbiota plays an important role in fermentation of non-digestible polysaccharides and results in the formation of health promoting metabolites e.g., acetate, propionate and butyrate [17]. It is believed that the ability of human gut microbiota to ferment non-digestible food components is not only limited to formation of health promoting metabolites but could also lead to the release of dietary toxins e.g., hydrolysis of masked mycotoxins [18].

Some animal studies [19,20,21] have observed modifications of the gut microbiota following mycotoxin exposure and suggest that there is a bi-directional interaction between mycotoxins and gut microbiota. However, the direct effect of mycotoxins on the growth of bacterial species from human colon has not been studied to date. The main bacteria capable of releasing mycotoxins in the human colon have also not been identified. This study is to our knowledge the first investigation assessing members of important groups and phyla of human gut bacteria for their individual activities towards masked and unconjugated mycotoxins. At the same time, the potential effect of mycotoxins on the growth of those gut bacteria was assessed.

## 2. Results

We screened 14 different bacterial strains from dominant groups of human gut microbiota for their ability to hydrolyse masked mycotoxins and to degrade unconjugated mycotoxins (Table 1). *Enterococcus mundtii* DSM 4838 was included as a positive control strain.

### 2.1. Bacterial Growth in the Presence of Mycotoxins

All bacterial strains grew well in 96-well plates using the media specified in Section 4.2. Growth curves were visually examined for all bacterial strains treated with low or high mycotoxin concentrations (2 or 10 nmol/mL) and no major differences were observed. Growth rates were also calculated (Table 2) and no significant differences were observed between mycotoxin-treated bacteria and controls.

**Figure 1:**
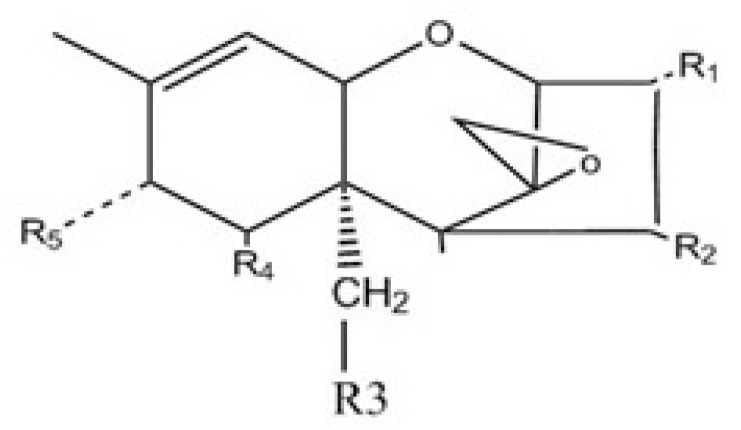
Chemical structure and ligands identifying the trichothecene mycotoxins used in this study and their masked forms and deacetylated metabolites.

The structure of mycotoxins and masked mycotoxins used and their deacetylated metabolites depicted in Figure 1.

### 2.2. Hydrolysis of Masked Trichothecenes by Bacterial Strains

Individual bacterial strains were anaerobically incubated with 2 nmol/mL of masked mycotoxins (DON-Glc or HT-2-Glc) or unconjugated mycotoxins (DON, DOM-1, HT-2, or T-2) for 48 h (Section 4.5) and mycotoxins analysed. The recovery of mycotoxins (masked forms + unconjugated forms) from bacterial cultures ranged from 80–120% for all bacterial strains except *B. fibrisolvens* 16/4 where DON recovery was low when incubated with DON-Glc (48% of dose added) or unconjugated DON (61% of dose added, data not shown). This could be partially due to bacterial surface binding of mycotoxins, which has been reported for strains of *Lactobacillus, Bifidobacterium*, and *Propionibacterium* binding ochratoxin A, aflatoxins, trichothecenes, and zearalenone in a strain-dependent manner [38,39,40]. All results were corrected for recovery and are presented as % of dose recovered to facilitate the comparison of hydrolysis efficiency between individual bacterial strains and different mycotoxins.

**Figure 2:**
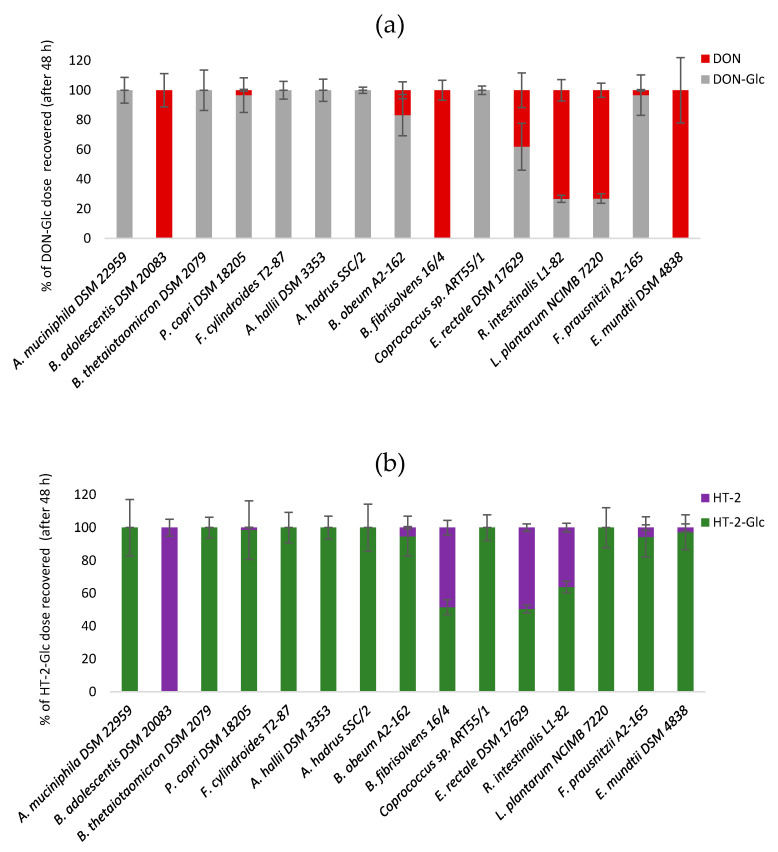
Hydrolysis of DON-Glc (panel **a**) and HT-2-Glc (panel **b**) by bacterial strains after 48 h incubation. Results are presented as percentage of the mycotoxin dose recovered and the average of triplicates ± SD.

Of the gut strains tested, *B. adolescentis* DSM 20083 and *B. fibrisolvens* 16/4 hydrolysed DON-Glc completely. Other strains partially hydrolysed DON-Glc (*P. copri* DSM 18205, *B. obeum* A2-162, *E. rectale* DSM 17629, *R. intestinalis* L1-82, *F. prausnitzii* A2-165, and *L. plantarum* NCIMB 7220; Figure 2a) to a varying extent while six strains tested negative for DON-Glc hydrolysis (Figure 2a). The positive control strain *E. mundtii* DSM 4838 was confirmed to hydrolyse DON-Glc completely within 48 h incubations.

Regarding the hydrolysis of the type A trichothecene HT-2-Glc, *B. adolescentis* DSM 20083 was found to be the most efficient strain, followed by *B. fibrisolvens* 16/4, *E. rectale* DSM 17629, and *R. intestinalis* L1-82. Minor hydrolysis of HT-2-Glc was also observed by *F. prausnitzii* A2-165, *B. obeum* A2-162, *P. copri* DSM 18205 and *E. mundtii* DSM 4838 while the remaining seven strains did not hydrolyse HT-2-Glc (Figure 2b). *B. adolescentis* DSM 20083 and *E. rectale* DSM 17629 hydrolysed DON-Glc and HT-2-Glc to a similar extent after 48 h while other strains (*B. fibrisolvens* 16/4, *R. intestinalis* L1-82, *L. plantarum* NCIMB 7220, *E. mundtii* DSM 4838) were more efficient in hydrolysing DON-Glc than HT-2-Glc.

### 2.3. Time-Course of Hydrolysis of Masked Trichothecenes by Selected Bacterial Strains

**Figure 3:**
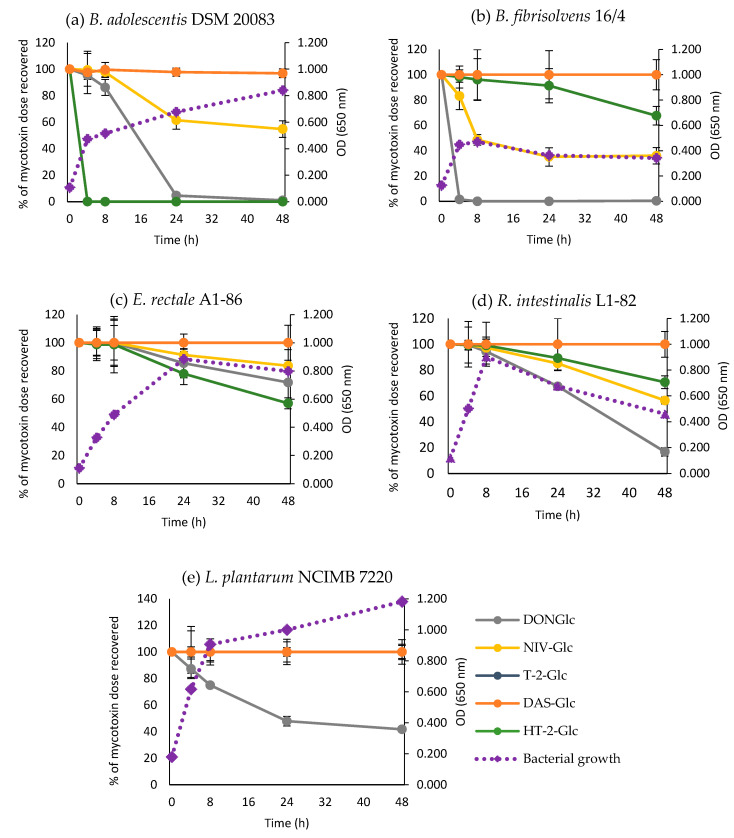
Time-course of hydrolysis of masked trichothecenes (solid lines) by (**a**) *B. adolescentis* DSM 20083, (**b**) *B. fibrisolvens* 16/4, (**c**) *E. rectale* A1-86, (**d**) *R. intestinalis* L1-82, (**e**) *L. plantarum* NCIMB 7220, and their growth (dashed lines) (**a**–**e**). Results are presented as percentage of the mycotoxin dose recovered and the average of triplicates ± SD (solid lines). T-2-Glc was only included for *L. plantarum* NCIMB 7220 (**d**). The SD for bacterial growth ranged between 0.01 and 0.05 per strain (dashed lines).

Based on the initial screening, mycotoxin hydrolysis was investigated in more detail in five strains. Time course experiments included time points 0, 4, 8, 24, and 48 h and further masked trichothecenes (NIV-Glc and DAS-Glc). *B. adolescentis* DSM 20083 was found to be the most efficient bacterial strain tested to hydrolyse masked mycotoxins (Figure 3a). This bacterium hydrolysed HT-2-Glc instantaneously with complete hydrolysis achieved after only 4 h incubation when bacterial growth was in the exponential phase. *B. adolescentis* DSM 20083 also hydrolysed the type B-trichothecenes DON-Glc and to a lesser extent NIV-Glc, mainly during late exponential phase of growth. *B. fibrisolvens* 16/4 on the other hand showed fastest hydrolysis of DON-Glc (during exponential growth phase) followed by NIV-Glc (during exponential to stationary phase) and HT-2-Glc (during stationary phase) (Figure 3b). *E. rectale* DSM 17629 was the least efficient strain tested in time-course experiments, only hydrolysing around 20% of masked mycotoxins after 24 h and 40% of HT-2-Glc after 48 h incubation (Figure 3c). Similarly, *R. intestinalis* L1-82 hydrolysed DON-Glc, NIV-Glc and HT-2-Glc in similar order but at a slower rate compared to *B. fibrisolvens* 16/4, with degradation mainly taking place during stationary phase (Figure 3b,d). *L. plantarum* NCIMB 7220 hydrolysed DON-Glc but did not break down any of the other trichothecene-glucosides tested (Figure 3e). None of the strains tested extensively hydrolysed the alpha-glucoside DAS-Glc.

**Figure 4:**
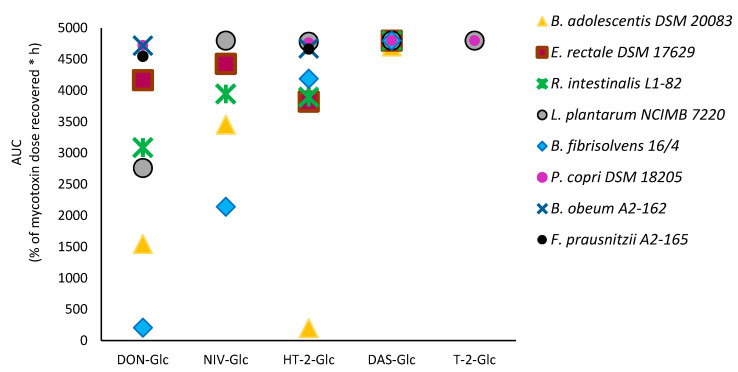
Difference in DON-Glc, NIV-Glc, HT-2-Glc, DAS-Glc, and T-2-Glc hydrolysis during 0–48 h by different human gut bacterial strains summarized by area under the curve (AUC).

The results from time-course experiments were used to calculate the area under the curve (AUC) for each masked trichothecene hydrolysed by each of the five bacterial strains. To allow complete comparison of active strains, AUC were also calculated for strains with minor hydrolysis activity using 0 and 48 h incubations (*L. plantarum* NCIMB 7220 hydrolysing HT-2-Glc; *F. prausnitzii* A2-165, *B. obeum* A2-162, and *P. copri* DSM 18205 hydrolysing DON-Glc and HT-2 Glc) (Figure 4). Lower AUC presents fast hydrolysis, whereas higher AUC shows slow or no hydrolysis of masked mycotoxins.

The two type B trichothecenes DON-Glc and NIV-Glc were hydrolysed by most of the bacterial strains tested with DON-Glc hydrolysis being most efficient (Figure 4). The type A trichothecene HT-2-Glc was efficiently hydrolysed by one bacterial strain, moderately hydrolysed by three and not altered by another. The type A alpha-glucoside DAS-Glc was not hydrolysed. This marked difference in hydrolytic capacity of individual bacterial strains and specific activity toward certain masked trichothecenes, but not others, will require further investigation. This paints a complex picture of very selective and specific interactions between bacterial strains and masked mycotoxins.

### 2.4. Degradation of Unconjugated Mycotoxins by Bacterial Strains

In addition to hydrolysis of masked mycotoxins by human gut bacteria, this study also assessed the potential microbial further metabolism of unconjugated mycotoxins (DON, T-2, HT-2, DAS, and NIV) in anaerobic culture for 48 h.

#### 2.4.1. De-Epoxydation of Trichothecenes

No de-epoxydation of DON to de-epoxy-deoxynivalenol (DOM-1) was observed by any of the strains tested and DON recoveries were ranging from 65.7 to 125.7% for all strains (data not presented). Mass fragments for de-epoxy NIV were also monitored but not detected. Similarly, HT-2 recoveries were also ranging from 78.7 to 113.4% for all strains (data not presented).

#### 2.4.2. De-Acetylation of Type A Trichothecenes

**Figure 5:**
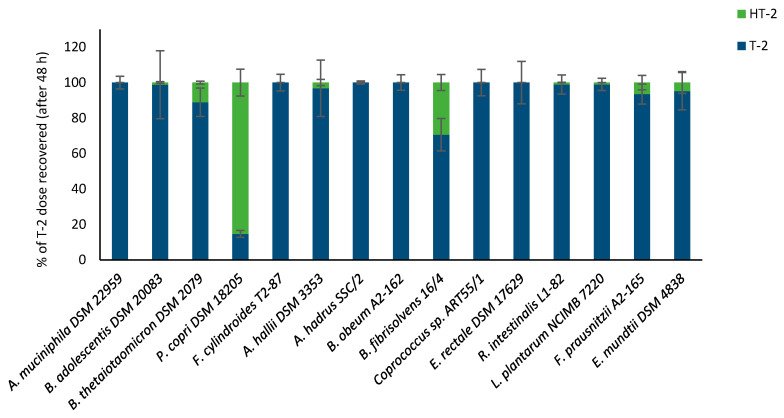
De-acetylation of T-2 by bacterial strains after 48 h incubation. Results are presented as a percentage of the mycotoxin dose recovered and the average of triplicates ± SD. Data are presented as T-2 degradation and HT-2 formation (0–48 h).

Out of the 15 bacterial strains tested, *P. copri* DSM 18205 was the most efficient strain in de-acetylating T-2 to HT-2 followed by *B. fibrisolvens* 16/4 (Figure 5). *L. plantarum* NCIMB 7220, *R. intestinalis* L1-82, *B. adolescentis* DSM 20083, *A. hallii* DSM 3353, *E. mundtii* DSM 4838, *F. prausnitzii* A2-165, and *B. thetaiotaomicron* DSM 2079 degraded minor amounts of T-2 to HT-2 (1.0–11.6%). The six remaining strains showed no activity towards T-2 after 48 h as no HT-2 was detectable and the recoveries for T-2 were between 90.9 and 119.5%.

**Figure 6:**
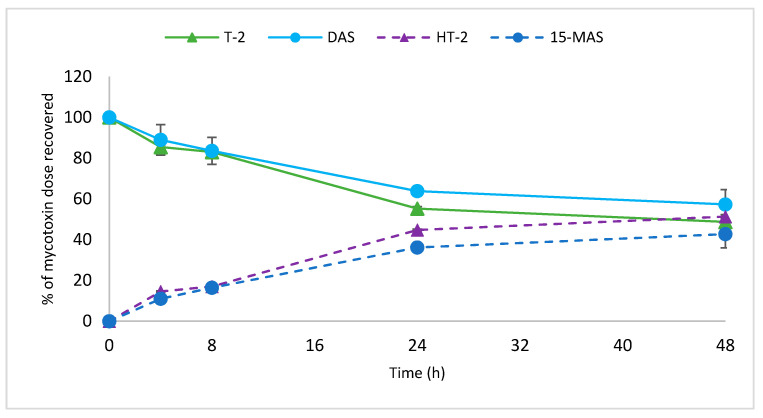
Time-course of de-acetylation of T-2 and DAS by *P. copri* DSM 18205. Results are presented as means of triplicate experiments ± SD.

De-acetylation of type A trichothecenes (T-2 and DAS) by *P. copri* DSM 18205 was confirmed in time course experiments, including time points 0, 4, 8, 24, and 48 h. *P. copri* DSM 18205 de-acetylated T-2 to HT-2 and DAS to 15-MAS at a slow rate (42.7 and 51.3%, respectively, after 48 h; Figure 6). The type A trichothecene alpha-glucosides DAS-Glc and T-2-Glc were not hydrolysed by *P. copri* DSM 18205 or any of the other strains tested (data not shown).

## 3. Discussion

The human gut microbiota is involved in the hydrolysis and metabolism of masked mycotoxins and unconjugated mycotoxins. However, the gut bacteria responsible remain poorly characterised. Here we describe the ability of individual strains of human gut bacteria to hydrolyse masked mycotoxins and further metabolise mycotoxins. Our results clearly demonstrate that under in vitro conditions, masked mycotoxins (DON-Glc, NIV-Glc, and HT-2-Glc) were converted to unconjugated mycotoxins (DON, NIV, and HT-2) upon incubation with different human gut bacterial strains with different hydrolysis rates. We observed that all the tested bacterial strains hydrolysed different masked mycotoxins in a strain-dependent manner, with differences in the rate of degradation. Some mycotoxins were completely hydrolysed during early exponential growth, whereas others were mostly degraded during stationary phase. For some incubations, hydrolysis plateaued before it was complete, for example *B. fibrisolvens* 16/4 ceased hydrolysis of NIV-Glc during stationary phase. The exact reason why bacterial activity decreases, or stops is unknown but one possible reason could be the depletion of bacterial resources to grow and hydrolyse masked mycotoxins in vitro. We have observed that *B. fibrisolvens* 16/4 switched their hydrolysis activity from masked forms of type B trichothecenes to masked forms of type A trichothecene after 24 h in stationary phase. This selective choice to hydrolyse different masked mycotoxins in different growth stages could be linked to their efficiency to hydrolyse masked mycotoxins and the structure of the masked mycotoxins.

These specific interactions between bacterial strains and individual mycotoxin-glucosides were also reported previously. Authors found the specific activity of a β-glucosidase enzyme purified from *B. adolescentis* to be highest towards DON-Glc (11 µmol/min/mg) compared to NIV-Glc and HT-2-Glc (0.18 and 3.5 µmol/min/mg, respectively), whereas our study found *B. adolescentis* DSM 20083 to be more efficient in hydrolysing HT-2-Glc than DON-Glc and NIV-Glc. However, Michlmayr et al. [18] used the substrates at varying concentrations (10, 1, and 2 mM for DON-Glc, NIV-Glc, and HT-2-Glc, respectively) all of which were higher than in the current study (2 µM) and they assessed enzyme preparations rather than whole bacterial cultures. *L. plantarum* NCIMB 7220 has been reported to be the most efficient bacterium to hydrolyse DON-Glc (62% hydrolysis after 8 h) followed by *E. mundtii* DSM 4838 and *B. adolescentis* DSM 20083 [41]. We confirm that *E. mundtii* DSM 4838 is very efficient in hydrolysing DON-Glc, whereas *L. plantarum* NCIMB 7220 and *B. adolescentis* DSM 20083 were slightly slower in hydrolysing DON-Glc compared to the published report. However, we started the bacterial incubations with less bacterial cells (OD_650nm_, 0.150 ± 0.050), compared to the previous study (OD_600_ about 2.0) [41].

In this study, none of the strains hydrolysed α-glucosides except minor hydrolysis of DAS-Glc by *B. adolescentis*. Therefore, the configuration of the glucosides (α and β linkages) is of importance along with the different types of masked mycotoxins. However, hydrolysis of α-glucosides has been reported in mixed human faecal samples [7,9,42] and further work is needed to identify the bacterial groups responsible for this activity.

We have identified bacterial strains from different groups of gut bacteria to be important contributors to mycotoxin hydolysis. In addition, we found *F. prausnitzii* A2-165, *P. copri* DSM 18205, and *B. obeum* A2-162 to release minor amounts of unconjugated mycotoxins from DON-Glc and HT-2-Glc. *F. prausnitzii* A2-165 belongs to the clostridial cluster IV and accounts for 5–10% of the total bacteria detectable in human faecal samples [43]. Similarly, *B. obeum* A2-162 is recognized as being abundant in the human colon [44] whereas *P. copri* DSM 18205 is present in high abundance only in a subsection of healthy Westerners [45]. Therefore, due to their high abundance in the human colon compared to our in vitro assays, it is suggested that these bacteria can significantly contribute to mycotoxin release in the human colon.

The gut bacterial strains we identified in the present study for masked mycotoxin hydrolysis contribute to gut microbiota composition in different proportions. *B. adolescentis* is abundant in the human colon [46]. *Bifidobacterium* species are more abundant in infants than in adults but comparatively stable in adults [47]. Our results demonstrate that *B. adolescentis* DSM 20083 can efficiently hydrolyse different masked mycotoxins and likely plays a vital role in hydrolysis of masked mycotoxins both in infants and adults. *L. plantarum* NCIMB 7220 was also found to efficiently hydrolyse DON-Glc, but had no activity towards other β-glucosides tested. *Lactobacillus* species usually only constitute ≤1% of the total bacteria in the human gut [48] and are more abundant in children. A recent study has proven that children are frequently exposed to multiple mycotoxins [49] and this high exposure is linked to the high intake of mycotoxins-contaminated cereals [50]. Therefore, it is likely children regularly ingest masked mycotoxins and ultimately their efficient hydrolysis due to the presence of *Lactobacillus* and *Bifidobacterium* species can increase their mycotoxin exposure.

*E. rectale* and *R. intestinalis* are among the most abundant firmicutes [51] as estimated to account for 5–10% of the total bacteria in the healthy human colon [43]. We observed that both strains were capable of hydrolysing both type A and type B trichothecenes, hence theses strains are major contributors to the hydrolysis of mycotoxin β-glucosides in the human colon. *Butyrivibrio spp.* have occasionally been reported in human faeces [33], but their presence is mostly linked to ruminants and other mammals [52]. In the present study we found that *B. fibrisolvens* 16/4 is very efficient in hydrolysing masked type B trichothecenes, suggesting that individuals harbouring this bacterium could be at higher risk of releasing unconjugated mycotoxins if ingesting masked mycotoxins through contaminated food.

Microbial metabolism of unconjugated mycotoxins could act as route of detoxification. A previous study [8] has reported the conversion of DON to DOM-1, a less toxic de-epoxydation metabolite [53], by human faecal microbiota. Similarly, de-acetylation of trichothecenes is considered a detoxification pathway for DAS [54,55] and DAS is de-acetylated by mixed human faecal microbiota [7]. In vitro studies also demonstrated that HT-2 is less cytotoxic than the T-2 [56,57]. The current study identified *P. copri* DSM 18205 as efficiently de-acetylating type A trichothecenes T-2 and DAS. *P. copri* is the most abundant *Prevotella* species in the human colon [58], comprising > 10% of relative abundance of *Prevotella* in faeces of 10–25% of healthy individuals from Europe and America [45]. The presence of *Prevotella* species in the human colon is mostly linked with vegetarian diets rich in plant fibre and carbohydrates [59,60,61,62]. Individuals harbouring *P. copri* DSM 18205 could therefore potentially decrease the mycotoxin load by converting mycotoxins into less toxic metabolites. *B. fibrisolvens* 16/4 can also de-acetylate T-2 (current study) and DAS [63], hence contributing to de-acetylation in the colon of individuals harbouring this bacterium.

Some in vivo studies have reported mycotoxin-induced modifications in gut microbiota of animals by using advanced molecular approaches. These authors reported that *Coprococcus* genus was more abundant in DON fed rats [20], whereas *Lactobacillus* was found more abundant in DON and ZEN fed pigs [21]. Another study in pigs observed that DON and ZEN in feed increased the relative abundance of Erysipelotrichaceae and decreased Ruminococcaceae, Streptococcaceae, and Veillonellaceae [19]. However, in the current study we observed that mycotoxin exposure (unconjugated or masked forms) did not impact the growth of any bacterial strain tested, suggesting that mycotoxins do not act directly on gut microbes. However, the large intestine and gut microbiota have been identified as targets of mycotoxin toxicity [64], but it is more likely that secondary effects on microbiota composition are caused by host-toxicity. More work is needed to further elucidate these interactions.

In summary, we have identified novel strains of gut bacteria likely to play a key part in hydrolysing masked mycotoxins in the human gut due to their high activity combined with high prevalence amongst the gut microbiota (*E. rectale* A1-86, *R. intestinalis* L1-82, and *B. fibrisolvens* 16/4) and confirmed the role of *B. adolescentis* and *L. plantarum*. We have also demonstrated that *P. copri* DSM 18205 is a key species in de-acetylation of type A trichothecenes and confirmed the activity in *B. fibrisolvens* 16/4. These findings support the notion that the microbial release of mycotoxins from their masked forms contributes to overall exposure in humans. The extent to which hydrolysis occurs varies in individuals, which likely depends on the individual composition of the gut microbiota and the use of probiotics (*Bifidobacterium* and *Lactobacillus* spp.) and prebiotics boosting the numbers of specific gut bacteria could influence the hydrolysis of masked mycotoxins in individuals.

## 4. Materials and Methods

### 4.1. Bacterial Strains

Thirteen strains of human gut bacteria were obtained from the Rowett Microbiology collection. *Akkermansia muciniphila* DSM 22959 and *Enterococcus mundtii* DSM 4838 were obtained from the German Collection of Microorganisms and Cell Cultures (DSMZ). *E. mundtii* DSM 4838 is a strain isolated from the human naval and was included as a control strain which was previously found to hydrolyse DON-Glc in vitro [41]. All strains were stored as glycerol stocks at −70 °C and revived overnight at 37 °C in M2GSC medium (+0.2% mucin for *A. muciniphila*) before culturing them in the respective growth medium (Table 1).

### 4.2. Medium Selection

All strains were grown in anaerobic yeast extract-casitone-fatty acids medium containing three substrates: glucose, starch, and cellobiose (YCFAGSC) [65], with the exception of *F. prausnitzii* A2-165 and *E. mundtii* DSM 4838, which were grown in anaerobic modified Med2 of Hobson (M2GSC) [66], and *L. plantarum.,* which was grown in anaerobic De Man, Rogosa and Sharpe medium (MRS broth, Sigma Aldrich, Gillingham, UK) for optimal growth.

### 4.3. Mycotoxin Standards

All mycotoxins used, their full names and abbreviations are summarized in Table 3. DON, DON-Glc, DOM-1, NIV, DAS, 15-MAS, T-2, and HT-2 were purchased from Romer Labs Ltd., Tulln, Austria. DAS-Glc, T-2-Glc [67] and HT-2-Glc [68] were obtained from Dr. Mark Busman and Dr Susan McCormick, Mycotoxin Prevention and Applied Microbiology Unit, USDA-ARS-NCAUR in the USA. NIV-Glc was obtained from Dr. Tomoya Yoshinari, National Institute of Health Sciences, Japan [69]. Working solutions for all mycotoxins were prepared in acetonitrile and stored at 4 °C.

### 4.4. Bacterial Growth and Mycotoxin Metabolism in Anaerobic 96-Well Plates over 48 h

Overnight cultures of all bacterial strains at early stationary phase were Gram-stained prior to each experiment. All bacterial and mycotoxin incubations were carried out in 96-well plates (Costar 3370, Corning Inc., Corning, NY, USA) and inside an anaerobic cabinet (Don Whitley MACS VA500, gas composition 10% hydrogen, 10% carbon dioxide, and balance nitrogen). Then, 4 µL of bacterial culture (in triplicate for each strain) were inoculated into 196 µL of medium (as specified in Section 4.2) containing either DON, DOM-1, DON-Glc, T-2, HT-2, or HT-2-Glc in acetonitrile (at 2 or 10 nmol/mL). The mycotoxin concentration of 2 nmol/mL was used to assess the hydrolysis/metabolism of masked or unconjugated mycotoxins by human gut bacteria in accordance with published studies [9,12]. The effect of mycotoxins on bacterial growth was assessed at 2 and 10 nmol/mL. Mycotoxin-free controls, solvent controls, and medium blanks as well as mycotoxin stability controls in medium blanks were included (in triplicates). The plates were sealed (Bio-Rad optical sealing tape, cat no 2239444), closed with a plate lid and placed into an EPOCH2 microplate reader (BioTek Instruments Inc. USA) and incubated anaerobically for 48 h at 37 °C. Growth was continuously monitored by automatically recording OD at 650 nm every 10 min, after 10 s of double orbital shaking (frequency 425 rpm, 3 mm). At the end of the experiment, growth data were captured using Gen 5 software [version 3.02.1]. Growth rates were determined as described by Soto-Martin et al. [70]. The exponential phase of growth (blank subtracted data) was visualised by plotting the OD reading on a logarithmic scale. Growth rates (µ/h) were calculated by choosing a time interval ensuring linearity from a trend line (*R*^2^ 0.99, with 5–10 data points).

At the end of the incubation, 180 µL of each bacterial suspension were transferred to Eppendorf tubes and the reactions stopped by adding 600 µL of acetonitrile. Samples were centrifuged at room temperature (10,000× *g*, 5 min). The supernatants were evaporated to dryness under a nitrogen stream at 50 °C, reconstituted in 1 mL of H_2_O and cleaned through pre-conditioned (2 mL of methanol twice followed by 2 mL of water) C18 solid phase extraction columns (Agilent, Wokingham, UK). Samples were eluted with 3 mL methanol, evaporated to dryness, and reconstituted in 180 µL of 50% aqueous methanol for LC-MS/MS analysis. Stability controls for each mycotoxin (in triplicate) were also included in each plate by adding target mycotoxins to the respective medium in the absence of any bacterial culture. All tested mycotoxins and their masked forms were found to be stable when incubated for 48 h in the absence of bacteria. For time 0 samples, overnight bacterial cultures were diluted 2/100 with their optimized anaerobic culture medium in Eppendorf tubes (200 µL), spiked in triplicate separately with 2 or 10 nmol/mL of DOM-1, DON, DON-Glc, T-2, HT-2, or HT-2-Glc in acetonitrile and processed immediately (time 0). Mycotoxins detected in time 0 samples were set as the reference (100%) and the results for 48 h time point were calculated as a percentage of time 0.

### 4.5. Time Course Experiments of Mycotoxin Metabolism of Selected Bacterial Strains

Initial screening experiments identified the following six bacterial strains which were further studied for mycotoxin metabolism: *Bifidobacterium adolescentis* DSM 20083, *Eubacterium rectale* A1-86, *Roseburia intestinalis* L1-82, *Butyrivibrio fibrisolvens* 16/4, *Lactobacillus plantarum* NCIMB 7220, and *Prevotella copri* DSM 18205.

Time course experiments were performed to compare the efficiency of different bacterial strains from the human gut to hyrdolyse masked mycotoxins. Each incubation was started with the same number of bacterial cells by diluting overnight cultures of each strain to an OD_650nm_ of 0.150 ± 0.050 in 96-well plates with anaerobic culture medium (total volume 200 µL). Each well was spiked separately with 2 nmol/mL of DON-Glc, NIV-Glc, HT-2-Glc, or DAS-Glc in acetonitrile (in triplicate). For microbial deacetylation, unconjugated mycotoxins T-2 or DAS were also tested in addition to their glucosides (*P. copri* DSM 18205 only). The plate was then sealed, closed with a lid, and inserted into the plate reader at 37 °C and OD (650 nm) measured every 10 min. At different time intervals (0, 4, 8, 24, and 48 h), the plate was removed from the plate reader and 180 µL of bacterial suspension from the appropriate wells were removed and the plate returned to the plate reader. Samples were transferred to Eppendorf tubes and further processed as described above (Section 4.5).

### 4.6. LC-MS/MS Analysis

The liquid chromatography analysis of the mycotoxins was performed on an Agilent 1200 HPLC system (Agilent Technologies, Wokingham, UK) fitted with an Agilent Zorbax 5 µm, 150 mm × 4.6 mm C18 column. For analysis of type A trichothecenes (T-2, T-2-Glc, HT-2, HT-2-Glc, 15-MAS, 4-MAS, SCP, DAS, and DAS-Glc) the mobile phase solvents were (A) 5 mM ammonium acetate and (B) methanol for all experiments. The starting gradients were 45% A and 55% B. The proportion of the 55% B rising linearly to 100% B at 13 min, held at 100% B for 2 min, with a 2.5 min column re-equilibration at 55% B. The flow rate was 400 µL/min, and the injection volume was 15 µL. For analysis of type B trichothecenes (DOM-1, DON, DON-Glc, deNIV, NIV, and NIV-Glc) mobile phase solvents were (A) 0.1% Acetic Acid and (B) methanol for all experiments. The starting gradient was 70% A and 30% B, the proportion of B rising linearly to 100% B at 15 min, held at 100% B for 2 min, with a 2.5 min column re-equilibration at 30% B. The flow rate was 350 µL/min, and the injection volume was 15 µL.

**Figure 7:**
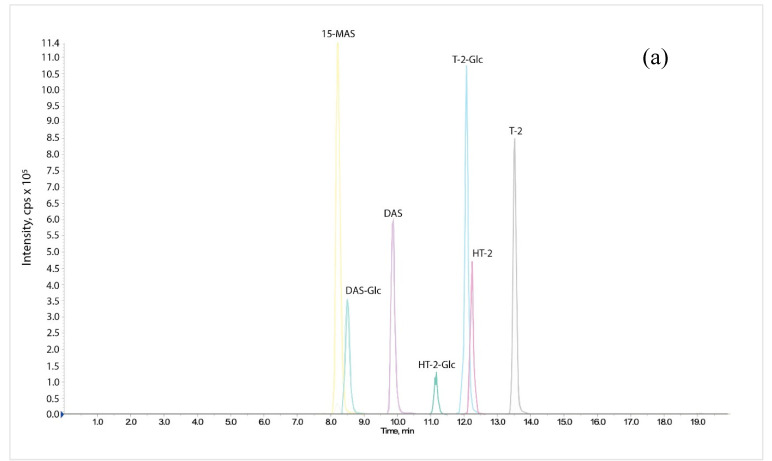

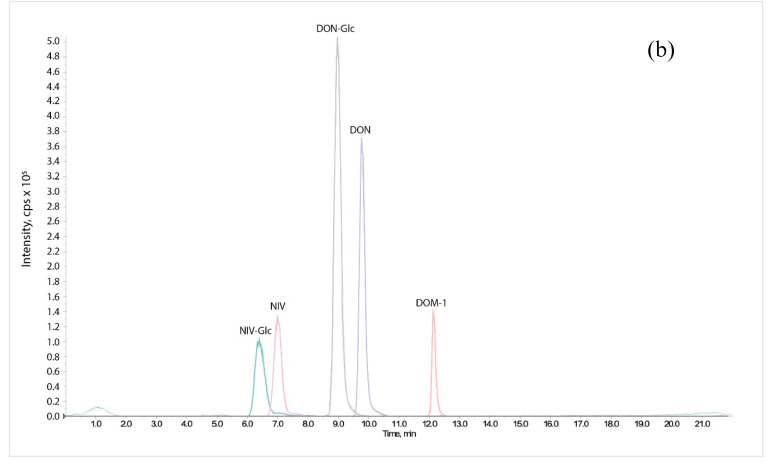
LC-MS/MS chromatograms for the quantification of type A trichothecenes (panel **a**) and type B trichothecenes (panel **b**) at 2 nmol/mL.

All mycotoxins were detected on a Q-Trap 4000 triple quadrupole mass spectrometer (AB Sciex, Warrington, UK) fitted with a Turbo Ion Spray™ (TIS) source. The mass spectrometer was run with the following source settings: ion spray voltage -4000 V, temperature 200 °C, Gases 1 and 2 set at 24 and 40 psi respectively and the Curtain Gas set at 10 psi in negative ion mode. Ion transition parameters are summarized in Table 3. Mycotoxins were quantified using the multiple reaction monitoring (MRM) technique. Standard solutions of 500 ng/mL were pumped directly into the TIS source via a syringe pump and their transition values were optimized. Calibration curves (0.25–10 nmol/mL equivalent to 74–2963 ng/mL DON, 115–4585 ng/mL DON-Glc, 70–2803 ng/mL DOM-1, 78 –3123 ng/mL NIV, 119–4745 ng/mL NIV-Glc, 117–4665 ng/mL T-2, 157–6287 ng/mL T-2-Glc, 106–4245 ng/mL HT-2, 147–5866 ng/mL HT-2-Glc, 92–3664 ng/mL DAS, 132–5286 ng/mL DAS-Glc, and 81–3244 ng/mL 15-MAS) were prepared for each metabolite, no internal standard was used. The precursor ions used were (M+Ac)− for DOM-1, DON, DON-Glc, NIV, and NIV-Glc and (M+NH4)^+^ for T-2, HT-2, DAS, 15-MAS and their glucosides. LC-MS/MS chromatrograms for all mycotoxin standards are presented in Figure 7.

### 4.7. Statistical Analysis

To explore the significant difference (*p* < 0.05) in growth rate of each bacterial strain across the different treatments, single step method was used in “R” (version 3.5.2 (2018-12-20)). Initially, a linear model without an intercept was conducted to calculate means across replicates of each treatment. The R package “multcomp” was used to construct a General Linear Hypothesis Test using the function “glht”. This function allows to specify several comparisons between treatments while adjusting for multiple testing. We used the single step method to correct for multiple testing which adjusts the *p*-values by using the joint t-distribution for the contrasts [71]. The contrast testing is similar to a t-test for comparing two groups with the difference that variance and standard error estimates are based on data from all groups involved in the analysis. The comparisons in this case were (^a^ 2 nmol/mL; ^b^ 10 nmol/mL):

1. Bacterial control vs. solvent control ^a^
2. Bacterial control vs. solvent control ^b^
3. Solvent control ^a^ vs. mycotoxin ^a^
4. Solvent control ^b^ vs. mycotoxin ^b^

## Figures and Tables

**Table:** 

Compound Name	Abbreviation	R1	R2	R3	R4	R5
T-2 toxin	T-2	OH	OAc	OAc	H	C_5_H_9_O_2_
HT-2 toxin	HT-2	OH	OH	OAc	H	C_5_H_9_O_2_
T-2-3-α,D-glucoside	T-2-Glc	C_6_H_11_O_6_	OAc	OAc	H	C_5_H_9_O_2_
HT-2-3-β,D-glucoside	HT-2-Glc	C_6_H_11_O_6_	OH	OAc	H	C_5_H_9_O_2_
4,15-Diacetoxyscirpenol	DAS	OH	OAc	OAc	H	H
15-Monoacetoxyscirpenol	15-MAS	OH	OH	OAc	H	H
4-Monoacetoxyscirpenol	4-MAS	OH	OAc	OH	H	H
Scirpenol	SCP	OH	OH	OH	H	H
DAS-3-α,D-glucoside	DAS-Glc	C_6_H_11_O_6_	OAc	OAc	H	H
Deoxynivalenol	DON	OH	H	OH	OH	=O
DON-3-β,D-glucoside	DON-Glc	C_6_H_11_O_6_	H	OH	OH	=O
Nivalenol	NIV	OH	OH	OH	OH	=O
NIV-3- β,D-glucoside	NIV-Glc	C_6_H_11_O_6_	OH	OH	OH	=O

**Table 1:** Summary of strains of human gut bacteria used in this study.

Phylum	Family	Bacterial Species	Reference(s)
Verrucomicrobia	Akkermansiaceae	*Akkermansia muciniphila* DSM 22959	[22]
Actinobacteria	Bifidobacteriaceae	*Bifidobacterium adolescentis* DSM 20083	[23]
Bacteroidetes	Bacteroidaceae	*Bacteroides thetaiotaomicron* DSM 2079	[24,25]
Bacteroidetes	Bacteroidaceae	*Prevotella copri* DSM 18205	[26]
Firmicutes	Erysipelotrichaceae	*Faecalitalea cylindroides* T2-87	[27]
Firmicutes	Lachnospiraceae	*Anaerobutyricum hallii* DSM 3353	[28,29]
Firmicutes	Lachnospiraceae	*Anaerostipes hadrus* SSC/2	[30]
Firmicutes	Lachnospiraceae	*Blautia obeum* A2-162	[27,31,32]
Firmicutes	Lachnospiraceae	*Butyrivibrio fibrisolvens* 16/4	[33]
Firmicutes	Lachnospiraceae	*Coprococcus sp.* ART55/1	[30,34]
Firmicutes	Lachnospiraceae	*Eubacterium rectale* DSM 17629 (A1-86)	[23]
Firmicutes	Lachnospiraceae	*Roseburia intestinalis* L1-82	[23,35]
Firmicutes	Lactobacillaceae	*Lactiplantibacillus plantarum* NCIMB 7220	[36]
Firmicutes	Ruminococcaceae	*Faecalibacterium prausnitzii* A2-165	[23]
Firmicutes	Enterococcaceae	*Enterococcus mundtii* DSM 4838 *	[37]

* *E. mundtii* was isolated from the human navel.

**Table 2:** Growth rates (µ/h) of bacterial strains in the presence of unconjugated or masked mycotoxins at two concentrations (2 or 10 nmol/mL).

Bacterial Strain	Bacterial	Solvent Control (nmol/mL)		DON (nmol/mL)		DON-Glc (nmol/mL)		HT-2 (nmol/mL)		HT-2-Glc (nmol/mL)	
	Control	2	10	2	10	2	10	2	10	2	10
*A. muciniphila* DSM 22959	0.148	0.197	0.191	0.210	0.202	0.131	0.141	0.128	0.127	0.122	0.150
*B. adolescentis* DSM 20083	0.747	0.708	0.732	0.728	0.715	0.619	0.622	0.764	0.656	0.642	0.647
*B. thetaiotaomicron* DSM 2079	0.099	0.157	0.107	0.085	0.108	0.110	0.100	0.097	0.089	0.121	0.096
*P. copri* DSM 18205	0.367	0.355	0.343	0.366	0.384	0.364	0.375	0.368	0.363	0.377	0.376
*F. cylindroides* T2-87	0.570	0.536	0.548	0.629	0.628	0.552	0.665	0.574	0.582	0.501	0.576
*A. hallii* DSM 3353	1.072	1.197	1.328	1.017	1.123	1.511	0.964	1.281	1.213	1.285	1.295
*A. hadrus* SSC/2	1.111	1.170	1.074	1.055	1.113	1.009	1.180	1.141	1.061	1.147	1.164
*B. obeum* A2-162	0.240	0.260	0.243	0.240	0.243	0.235	0.261	0.251	0.264	0.255	0.268
*B. fibrisolvens* 16/4	0.254	0.237	0.317	0.220	0.290	0.265	0.292	0.271	0.289	0.298	0.367
*Coprococcus sp.* ART55/1	0.775	0.834	0.852	0.782	0.803	0.696	0.941	0.781	0.678	0.626	0.551
*E. rectale* DSM 17629	0.301	0.308	0.328	0.290	0.333	0.317	0.340	0.337	0.357	0.345	0.358
*R. intestinalis* L1-82	1.229	1.178	1.141	1.208	1.188	1.154	1.153	1.156	1.172	1.222	1.120
*L. plantarum* NCIMB 7220	0.731	0.733	0.747	0.768	0.800	0.784	0.769	0.772	0.765	0.773	0.781
*F. prausnitzii* A2-165	0.523	0.552	0.531	0.559	0.526	0.539	0.546	0.523	0.503	0.500	0.453
*E. mundtii* DSM 4838	1.325	1.286	1.379	1.273	1.381	1.560	1.361	1.371	1.347	1.281	1.286

Data are presented as the average of triplicate growth rate measurements and SD ranged between 0.01–0.30 for all measurements.

**Table 3:** Summary of mycotoxin metabolites and their ion transition parameters used in LC-MS/MS analysis.

Compound	Precursor Ion (*m*/*z*)	Product Ion (*m*/*z*)	Retention Time (RT) (min)	Dwell Time (msec)	Collision Energy	Polarity
DOM-1	339.1	249.10	11.64	75	−16.0	Negative
DON	355.1	265.10	9.47	75	−21.0	Negative
DON-Glc	517.3	427.30	8.65	75	−29.0	Negative
NIV	371.1	281.1	6.76	75	−21.5	Negative
NIV-Glc	533.3	473.4	6.16	75	−19.5	Negative
deNIV	355.1	265.1	6.96	75	−21.5	Negative
T-2	484.4	185.3	12.87	50	30.5	Positive
T-2-Glc	646.4	305.1	11.76	50	26.5	Positive
HT-2	442.3	215.3	11.60	50	17.0	Positive
HT-2-Glc	604.4	323.1	10.53	50	17.0	Positive
DAS	384.4	307.3	9.31	50	17.0	Positive
DAS-Glc	546.3	349.3	7.99	50	20.5	Positive
15-MAS	342.2	265.2	7.80	50	12.0	Positive
4-MAS	342.2	217.1	5.90	50	17.0	Positive
SCP	300.2	247.1	5.3	50	17.0	Positive

DOM-1, Deepoxy-deoxynivalenol; DON, Deoxynivalenol; DON-Glc, DON-3-β,D-glucoside; deNIV, Deepoxy-nivalenol; NIV, Nivalenol; NIV-Glc, NIV-3-β,D-glucoside; T-2, T-2 toxin; T-2-Glc, T-2-3-α,D-glucoside; HT-2, HT-2 toxin; HT-2-Glc, HT-2-3-β,D-glucoside; DAS, diacetoxyscirpenol, DAS-Glc, DAS-3-α,D-glucoside; 15-MAS, 15-monoacetoxyscirpenol; 4-MAS, 4-monoacetoxyscirpenol; SCP, scirpenol.
